# Sexual Function and Insanity

**Published:** 1900-09-29

**Authors:** 


### Hospital Clinics and Medical Progress.

### SEXUAL FUNCTION AND INSANITY.

A subject full of importance to the alienist, but even more so to the general practitioner, is the relation between sexual function, insanity, and crime. To the alienist who has his patients within the four walls of an asylum the question whether or no he may be able to give relief by operative treatment is constantly arising, but, interesting as it is, its solution is not a matter of immediate concern. At the worst he can wait and see what time will bring forth. To the general practitioner, however, the problem takes another form, for it is often a question requiring instant decision, whether a patient ought to be sent straightway to an asylum, or whether by waiting until the little cloud of sexual irritability blows over the mental crisis also will be recovered from. There are those no doubt who, regarding mind as the great distinguishing attribute of man, protest, notwithstanding the evidence of their senses, against the "materialistic" suggestion that mental processes are directly swayed by bodily conditions. Such people are still more aggrieved at the notion that mind can be the slave of that instinct which, above all others, we share with the brutes, namely, that of reproduction—the sexual function. Yet if one regards the general scheme of animal life as seen on every side, and notes in how large a degree it culminates in reproduction, often ending as soon as this is accomplished, and very commonly changing its manifestations with the cessation of the reproductive capacity, one would indeed have cause for wonder if the highest manifestations of nervous action, namely, the operations of mind, were to run their course independent of and undisturbed by that function which is the climax of the bodily activities, including those of nervous tissues of every kind. That sex and sexual function are predominant influences, by which the whole mental life of the healthy individual is guided, and to which is due much of the world's progress, is a fact which has been recognised by thinkers and by writers from the earliest times, and there would seem to be every *à priori* reason for believing that unsound minds are swayed even more than sound ones by the same all-powerful mainspring.

At the annual meeting of the British Medical Association Dr. Macnaughton-Jones introduced a discussion on the Correlation Between Sexual Function, Insanity, and Crime. He considered the matter both from the side of the gynæcologist and that of the alienist, and pointed out that the rhythmic cycle which month after month terminates in the discharge of the ovum is connected not only with considerable alteration in the ovarian, uterine, and broad ligament vascular and nervous supplies, but also with a condition of nervous exaltation in which reflex action is readily excited and morbid reflexes generated, attended possibly by their corresponding psychic states. Turning more especially to the question as it affects women, he said that there were two periods of life during which this rhythmic cycle of activities specially affected the brain and nervous system. First, at puberty and during adolescence any deviation from the normal balance of healthy nutrition in the tissues of the body was attended by loss of inhibitory power and nerve control, by greater susceptibility to exaggerated reflexes, morbid subjective sensations and perverted emotions, and by disturbances of all the reproductive and developmental attributes which the growing woman would present socially, intellectually, and morally to her environment. The occurrence of epilepsies, choreas, suicidal leanings, delusional insanity, and distorted or exaggerated sexual impulses were evidence of such loss of inhibition. Again, when the sexual life of the woman was approaching its close, and at a period when changes were occurring in the sexual organs—changes which had their accompanying psychical expressions, the blushings, the altered temper, the moroseness, the caprices of the menopause—we had like mental losses of inhibitory power and of nerve control. So we found at this time the various forms of climacteric insanity, with frequent suicidal impulses and delusional states, mixed up with perverted sexual ideas of self, as well as others relating to the married state. It is to be noted, however, that such manifestation of mental disturbance need not be regarded as directly due to the sexual disturbance, but rather as resulting from the fact that at both these periods of her life the patient is apt, in consequence of her lessened inhibitory power, to display those temperamental traits which she has inherited. The mental factors which can already in early childhood be recognised as likely to dominate the character of the future adult are more fully evolved and finally developed during the years of adolescence, and while they may in the intervening years of adult life be partially held in abeyance, they are apt to again manifest themselves during the years when that cessation of the activity in the reproductive organs takes place, which is one of the early signs of general decadence in the female organism.

We see then that there are two periods of life at which the mind is less under control than at other times (if we may permit ourselves to employ for the moment the more or less popular conception of an upper and a lower brain—a controlling and a controlled mind). But the repeated rhythmic disturbance set up by menstruation acts in the same way. Dr. Macnaughton-Jones says: "The clinical evidence is overwhelming that the simple physiological discharge of the function of ovulation, even where there are no symptoms of disturbance, or any pointing to abnormal conditions, has in some women a distinct psychic effect, amounting in a few to obvious mental excitability or depression, slight changes in temper, irritability, or moroseness, or hysterical symptoms. It is, however, when we come to consider the consequences of the various forms of erratic menstruation that we find different degrees of mental disturbance in their more aggravated forms." Summing up the whole matter, he concludes that: (1) Functional disorders of ovulation are frequently attended by mental aberration, and in a proportion of cases originate the mental disturbance, the same remark applying also to disorders of ovulation which have a pathological cause. (2) In the great majority of such cases the nervous disturbance is of a neurasthenic character, and is associated with various visceral or other neuroses. In only a small proportion does the alienation assume so grave a type as melancholia, mania, or dementia. (3) Where in an insane person ovulation and its extenal manifestation, the menstrual discharge, are erratic or absent, this may be a consequence of the general and insane condition, and not a causal factor in its production. Nevertheless, such abnormal menstruation appears to have an aggravating effect on the insanity, and there is sufficient evidence to support the view that—especially if due to a pathological cause—it should be treated. (4) The question of a gynæcological examination of an insane woman must be a matter for the discretion of the psychologist. As a universal practice it is not advisable. (5) Sufficient evidence is now advanced to justify the removal of the adnexa or uterus in insane women, when there are gross lesions of the former or tumours of the latter. (6) It does not appear that there is in healthfully-minded women who suffer from disease of the genitalia any special risk of post-operative insanity. On the other hand, if there be a psychopathic predisposition which had existed prior to and independently of the sexual disease, there is in such cases a larger percentage of post-operative mental disturbance than follows other operations. In such cases the prudence of radical operation may have to be discussed: but post-operative mental effect does not appear generally to be of a serious or permanent nature. (7) It may be generally affirmed that when mental disease of a graver type follows upon sexual disorder there has been an underlying and often unrecognised psychopathic predisposition, the disorder of the genitalia completing the circle needful for the final manifestation of the mental condition. (8) The relation of aberrant sexual function, or a disorder of menstruation, to any criminal act ought to be taken into consideration in determining the responsibility of the woman.

The conclusions put forward by Dr. Macnaughton-Jones appear moderate and reasonable, and it will be noted that they do not go nearly so far as those advocated by some gynæcologists. As illustrating the relation between mental disturbance and the periods which are mentioned above as being those of greatest instability, it may be mentioned that Dr. Wynn Westcott said that having held inquests on 700 suicides, more than 200 of these being women, he found that the majority of these women had killed themselves about the change of life, and that of the younger women, the majority appeared to have been menstruating at the time of the suicide.

An interesting observation was also made by Mr. Cardew, who said that, as medical officer of large schools for girls, his experience was that amenorrhœa was very common amongst the senior classes. As a rule, these girls were free from anæmia and hysteria, and the amenorrhœa disappeared in the holidays. There was, he said, no tendency for this amenorrhœa to produce any mental disturbance; he had only seen such a tendency manifested in one or two cases, which, of course, were immediately removed. The advocates of advanced education for growing girls will doubtless take this to mean that mental work does such girls no harm. The fact here vouched for, that in healthy girls the menses are apt to be stopped by study, even in the absence of anæmia, may, however, be taken to bear another explanation, and it seems a somewhat serious point against pushing mental effort at a time when it is above all things important that the proper periodicity of a woman's life should become fully established.

